# Resveratrol loaded polymeric micelles for theranostic targeting of breast cancer cells

**DOI:** 10.7150/ntno.51955

**Published:** 2021-01-01

**Authors:** Yiota Gregoriou, Gregoria Gregoriou, Vural Yilmaz, Konstantinos Kapnisis, Marianna Prokopi, Andreas Anayiotos, Katerina Strati, Nikolas Dietis, Andreas I. Constantinou, Chrysafis Andreou

**Affiliations:** 1 Department of Biological Sciences, Faculty of Pure and Applied Sciences, University of Cyprus, Nicosia, Cyprus.; 2 Department of Electrical and Computer Engineering University of Cyprus, Nicosia, Cyprus.; 3 Emphasis Research Centre, University of Cyprus, Nicosia, Cyprus.; 4 Department of Mechanical Engineering and Material Science and Engineering, Cyprus University of Technology, Limassol, Cyprus.; 5 Medical School University of Cyprus, Nicosia, Cyprus.

**Keywords:** cancer nanomedicine, nanotheranostics, resveratrol, breast cancer, drug-delivery

## Abstract

Treatment of breast cancer underwent extensive progress in recent years with molecularly targeted therapies. However, non-specific pharmaceutical approaches (chemotherapy) persist, inducing severe side-effects. Phytochemicals provide a promising alternative for breast cancer prevention and treatment. Specifically, resveratrol (res) is a plant-derived polyphenolic phytoalexin with potent biological activity but displays poor water solubility, limiting its clinical use. Here we have developed a strategy for delivering res using a newly synthesized nano-carrier with the potential for both diagnosis and treatment.

**Methods:** Res-loaded nanoparticles were synthesized by the emulsion method using Pluronic F127 block copolymer and Vitamin E-TPGS. Nanoparticle characterization was performed by SEM and tunable resistive pulse sensing. Encapsulation Efficiency (EE%) and Drug Loading (DL%) content were determined by analysis of the supernatant during synthesis. Nanoparticle uptake kinetics in breast cancer cell lines MCF-7 and MDA-MB-231 as well as in MCF-10A breast epithelial cells were evaluated by flow cytometry and the effects of res on cell viability via MTT assay.

**Results:** Res-loaded nanoparticles with spherical shape and a dominant size of 179±22 nm were produced. Res was loaded with high EE of 73±0.9% and DL content of 6.2±0.1%. Flow cytometry revealed higher uptake efficiency in breast cancer cells compared to the control. An MTT assay showed that res-loaded nanoparticles reduced the viability of breast cancer cells with no effect on the control cells.

**Conclusions:** These results demonstrate that the newly synthesized nanoparticle is a good model for the encapsulation of hydrophobic drugs. Additionally, the nanoparticle delivers a natural compound and is highly effective and selective against breast cancer cells rendering this type of nanoparticle an excellent candidate for diagnosis and therapy of difficult to treat mammary malignancies.

## Introduction

Breast cancer therapy with molecularly targeted approaches has evolved significantly in the past few years, however, not all patients could benefit from such approach. As defined via gene expression analysis, breast cancer is not a homogeneous disease but rather a combination of unique and distinctive subtypes 1. These subtypes are characterized by the expression of hormone receptors like the estrogen receptor (ER) and the progesterone receptor (PgR), and the growth factor receptor HER2. This trio of cell-surface receptors are often exploited for the targeting of “magic bullet” drugs to the cancerous tissue. In the absence of these receptors targeting becomes very problematic and results in poor prognosis and treatment 2.

Triple negative breast cancer (TNBC) is a specific basal like subtype which is negative for all three markers - ER, PgR, and HER2 3,4 and accounts for 15-20% of all breast cancer cases 5. Due to TNBC's unique biology, hormonal and anti-HER2 therapies are ineffective, rendering this type of cancer clinically challenging. Additionally, TNBC is highly aggressive and highly metastatic 5. Currently, the only available treatments for TNBC patients are limited to chemotherapy, radiation and surgery 6,7. In current treatments, chemotherapy is the most employed choice. Although good chemotherapeutic agents exist, most fail to differentiate between healthy cells and malignant cells, resulting in systemic toxicity and severe side effects. It is therefore critical to develop more effective and less toxic strategies for the treatment of breast cancer.

Phytochemicals have been employed in breast cancer prevention and treatment with promising results 8. Resveratrol (res), (trans-3,4',5-trihydroxystilbene, C_14_H_12_O_3_) is a plant-derived polyphenolic phytoalexin with potent biological activity. It is a non-flavonoid polyphenol found in abundancy in the skin of red grapes but has also been identified in other plant sources such as berries, soy beans, pomegranate and peanuts. Res has presented an array of medical benefits due to its interaction with a number of molecular targets 9-12. For example, res found in red wine has been notoriously linked to the 'French paradox' for its cardioprotective effects. Besides its cardioprotective effects res has also been examined for its anticancer properties. Consequently, there is considerable evidence in the literature for the use of res as a chemopreventive and chemotherapeutic agent against various types of malignancies, including breast cancer 13-19. Res's potential against mammary carcinogenesis has been well established in the literature and has been linked to various cell signaling pathways which cause cell cycle arrest, induce apoptosis, suppress proliferation, reduce inflammation and angiogenesis, and inhibit metastasis 20,13,15. Furthermore, res behaves as a phytoestrogen and has been utilized in hormone-dependent therapy against ERα and PR subtypes of breast cancer 21. Similarly, it has been shown that res enhances the cytotoxic efficacy of commonly used chemotherapeutic drugs 22 such as doxorubicin, in the treatment of a range of cancers 23. Despite the abundance of literature supporting the use of res as a potent therapeutic agent, res exhibits poor clinical performance. This is most likely because res displays poor water solubility, short half-life and chemical instability *in vivo*. Consequently, achieving adequate bioavailability at an effective therapeutic dose in clinical studies has been the main impediment towards its clinical use 24-26.

Nano drug delivery systems have been utilized to circumvent the limitations associated with hydrophobic drug molecules like res 27. Nanomaterial-based carriers offer a solution to problems associated with stability, solubility and toxicity of pharmaceuticals by offering protection from degradation, enabling controlled release and biodistribution and by increasing bioavailability through specific targeting. The development of biodegradable, polymer based nanocarriers, specifically ones prepared from amphiphilic block copolymers, has gained considerable attention in recent years. Polymeric micelles are a promising approach as they spontaneously self-assemble into nano-sized constructs. Normally, amphiphilic block copolymers composed of hydrophilic and hydrophobic segments can self-assemble into polymeric micelles at a concentration above their critical micelle concentration (CMC) 28-30.

In this work, we present an optimized method for preparing a nanodelivery system for breast cancer made from pluronic F127 block copolymer and using Vitamin E TPGS as the emulsifier for the delivery of res *in vitro*. This nanoparticle is unique because it comprises of a combination of biodegradable and biocompatible materials with known anticancer properties. Moreover, the same type of nanoparticle is also used to carry a fluorophore, Coumarin 6, offering the convenience of simultaneous diagnosis and therapy into a single nanoplatform 31-34.

**Figure 1:**
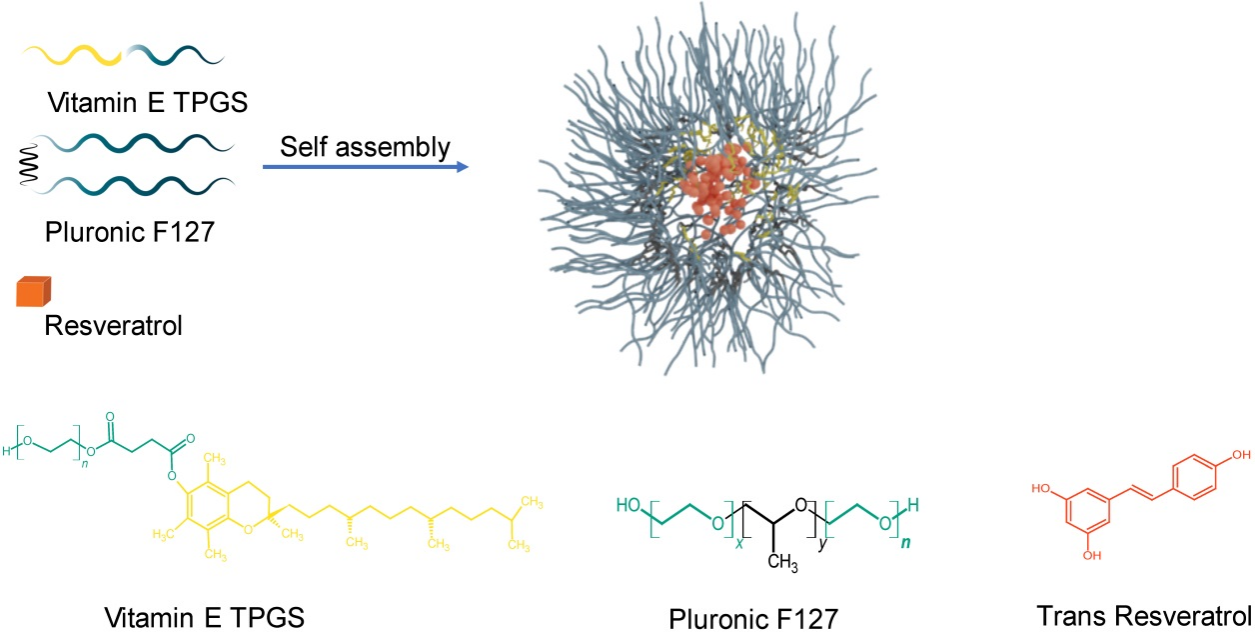
**Schematic illustration for the preparation of resveratrol loaded nanoparticles.** Hydrophilic regions for both Vitamin E TPGS and Pluronic F127 are represented by aqua blue and form the outside layer of the nanoparticle. The hydrophobic regions of Vitamin E TPGS and Pluronic F127 are coloured yellow and black, respectively and form the inner core. Resveratrol is encapsulated in the inner core and is illustrated by a brick colour. Chemical structures of D-*α*-tocopheryl polyethylene glycol 1000 succinate (Vitamin E TPGS), Pluronic F127 (x is 100 and y is 65) and trans-Resveratrol are shown.

Pluronic F127 is an amphiphilic triblock copolymer composed of poly (ethylene oxide) (PEO) and poly (propylene oxide) (PPO), PEO-*x*-PPO-*y*-PEO-x). Amphiphilic block copolymers self-assemble spontaneously in aqueous environments into polymeric nanostructures known as micelles and for this reason they have been commonly used to solubilize hydrophobic drugs in drug delivery. Shown schematically in **Figure 1**, the PEO block of F127 is hydrophilic and forms the outside layer of the nanoparticle while the PPO block is hydrophobic and composes the inner core. The hydrophobic core serves as a reservoir in which the hydrophobic drug molecule can be incorporated and protected from inactivation in biological media so it can be delivered effectively to the malignancy while the hydrophilic shell promotes the delivery of the drug to target cells 35,28,36. Pluronic F127 has attracted a lot of attention in drug delivery because of its low toxicity in the body and the ability to encapsulate hydrophobic agents. Additionally, Pluronic F127 enhances pro-apoptotic signalling, thereby sensitizing tumour cells and making them more vulnerable to the effects of anticancer drugs 31,33,34,37,38. Likewise, Vitamin E TPGS, a synthetic derivative of natural alpha-tocopherol, is an FDA and EFSA approved pharmaceutical adjuvant, frequently used in the development of DDS to improve the pharmacokinetics of anti-cancer drugs and reduce multi-drug resistance 39-43. Additionally, Vitamin E TPGS has been shown to greatly enhance the performance of nanoparticles, resulting in much higher cellular uptake of the drug as well as more desirable *in vivo* pharmacokinetics 44.

In this study, the proposed nanoformulation was synthesized and subsequently characterized by particle size, morphology, encapsulation efficiency (EE) and drug loading (DL) content. Additionally, the formulation's performance as a diagnostic and therapeutic agent was evaluated by a cellular uptake assay and an *in vitro* cell viability assay using breast cancer cell lines MCF-7 and MDA-MB-231 as well as with immortalized MCF-10A breast epithelial cells. The nanoparticle showed an enhanced res EE of 73% and DL content of 6.2%. Additionally, the nanoparticle showed superior uptake in breast cancer cells compared to control epithelial cells. Importantly, the nanoparticle reduced the viability of MCF-7 and MDA-MB-231 breast cancer cells with no effect on MCF-10A, rendering this nanoparticle a good candidate for diagnosis and therapy of difficult to treat mammary malignancies.

## Methods

### Materials

Res was purchased from Selleck chemicals (Dallas, TX., USA). The purity as determined by HPLC was 99.73%. Pluronic F127 was purchased from Sigma-Aldrich (St. Louis, MO., U.S.A), coumarin 6 (MW 350.43) from Santa Cruz Biotechnology (Dallas, TX, USA) and D-Alpha Tocopheryl Polyethylene Glycol 1000 Succinate (Vitamin E TPGS) from Eastman Chemical Company (Kingsport, TN, USA). Acetone (ACS grade) and Dichloromethane anhydrous (purity ≥ 99.8 %) were purchased from Sigma-Aldrich and Dimethyl Sulfoxide, extra pure (99.9%) from Scharlau chemicals (Barcelona, Spain).

### Preparation of the nanoparticle

Res-loaded nanoparticles were prepared by the single-emulsion method. The preparation method comprises mixing Pluronic F127 (EO_106_PO_70_EO_106_, MW=12,600) with dichloromethane as the solvent and D-alpha-tocopheryl polyethylene glycol 1000 succinate (Vitamin E TPGS) as the emulsifier. More specifically, 100 mg of Pluronic F127 were mixed in 1 mL of dichloromethane and 50 μl of res in acetone (at a concentration of 50 mg/mL) were added to the polymer mixture by vortexing until the encapsulant was homogeneously dispersed. The polymer/encapsulant solution was then added swiftly to Vitamin E TPGS at a ratio of 1:2 on high vortex. The resulting oil-in-water emulsion was ultrasonicated (Misonix, Ultrasonic Liquid Processors) in three 10 sec bursts (50% amplitude) on ice to induce nanosized droplets. The emulsion was then transferred into a beaker containing 45 mL of Vitamin E TPGS and stirred for 3 h to evaporate the solvent. The resulting hardened nanoparticles were then recovered by centrifugation on a fixed rotor at 17,000 rcf for 15 min at 22°C (Cientec CT-15000R centrifuge). The nanoparticles were washed 3 times via centrifugation with distilled water to remove any unentrapped drug. The supernatant containing unentrapped drug and unused polymer was collected and further tested to determine the drug entrapment efficiency of the nanoparticle. The resulting nanoparticle suspension was transferred to 15 mL pre-weighed falcon tubes, cooled to -80 °C for 3 hours and freeze-dried (Mecha Tech) for 48 hrs. For coumarin 6 loaded nanoparticles, coumarin 6 was added as the drug in place of res. Coumarin 6 was added directly to the polymer solution at a polymer to drug ratio of 1:40 by mass. The fabrication steps following were performed in the same manner as in the case of res-loaded nanoparticles and as mentioned above. The freeze-dried nanoparticles were wrapped in foil and stored at 4°C until further use. The freeze-dried nanoparticles were resuspended in ultrapure water (Sartorius Arium 611 VF Water Purifier system) and the solution was sonicated in a water bath sonicator (RS Pro, ultrasonic cleaner) three times for 15 min each time and subsequently filtered through a 0.45 μm syringe filter (PTFE, Hydrophilic, Dissolution Accessories, (Oosterhout, The Netherlands)) to remove all aggregates and obtain nanoparticles of uniform size prior to use.

### Analysis of size and morphology

#### Scanning Electron Microscopy

Morphological examination was performed with Scanning Electron Microscopy (SEM). Samples were mounted on aluminum specimen stubs and gold-sputtered to 5 nm thick films to prevent beam charging effects (SC7640 Sputter coater, Quorum Technologies, Kent, UK). High resolution scanning electron microscopic analysis was performed at 20 kV (magnification range of 30,000-120,000×) using a FEI Quanta 200 (FEI, Oregon, USA) microscope and images were processed using the ImageJ software.

#### Tunable Resistive Pulse Sensing (TRPS) size analysis

Quantification and size analysis of nanoparticles was performed using the qNano Gold platform (Izon Science, Oxford, U.K.). Nanoparticles were diluted in filtered PBS, measured using the nanopore NP200 (Izon Science, Oxford, U.K.) and compared to calibration particles CPC200. Data analysis was carried out using the Izon Control Suite software v3.3 (Izon Science, Oxford, U.K.).

#### Composition analysis by UV-Vis

The composition of the nanoparticles was verified by UV-Vis spectrophotometry (Perkin Elmer Lambda 1050, USA). For this purpose, the UV-Vis spectra of pure res, pure Pluronic F127, pure Vitamin E TPGS, res-loaded-nanoparticles and empty nanoparticles were attained. The samples were scanned at a wavelength of 250-850 nm. All solids and lyophilized nanoparticles were dissolved in DMSO prior to analysis.

#### Determination of the Encapsulation Efficiency and Drug loading content

The percent Encapsulation Efficiency (EE%) and the percent Drug Loading (DL%) content of res-loaded nanoparticles was determined by analysis of the supernatant obtained from the washes during synthesis (un-encapsulated drug) against a standard calibration curve of pure res. A representative 2 mL sample from each wash was centrifuged at 10,000 r.p.m. for 10 min at room temperature. The supernatant was collected and analysed on a multimodal microplate reader (Tecan Spark 20M) to measure the fluorescence intensity of the sample (excitation wavelength of 356 nm and an emission wavelength of 383 nm). For the calibration curve pure res was first diluted in DMSO to form a stock solution. This was further diluted in PBS to form a series of concentrations for the calibration curve. All experiments were performed in triplicates.

The % entrapment efficiency (EE%) is calculated from equation (1) 45:

EE% = (C_1_/C_2_) ×100% (1)

The % drug loading content (DL%) is calculated from equation (2) 45:

DL% = C_1_/(C_2_+C_3_) ×100% (2)

where C_1_ is the amount of res encapsulated in res-loaded NPs, C_2_ is the total amount of res used during synthesis and C_3_ is the total amount of polymer used during synthesis.

### Cell culture and Reagents

MCF-7, MDA-MB-231 and MCF-10A cell lines were obtained from the American Type Culture Collection (ATCC) (Manassas, VA). MCF-7 and MDA-MB-231 breast cancer cell lines were cultured in DMEM supplemented with 10% fetal bovine serum (FBS) and 1% antibiotic/antimycotic. MCF-10A immortalized breast cell line was cultured in DMEM F12 supplemented with 20 ng/mL EGF, 100 ng/mL Cholera Toxin, 500 ng/mL Hydrocortizone, 10 μg/mL Insulin, 5% Horse Serum (HS) and 1% antibiotic/antimycotic. Sub culturing of the cells was performed using 0.25% trypsin. The RPMI, FBS, antibiotics and trypsin used in cell culture were purchased from Gibco, Invitrogen (Carlsbad, California, USA).

### *In vitro* cellular uptake

#### Flow cytometry

Considering that res is a non-fluorescent drug, coumarin 6 was incorporated as a fluorescent dye during nanoparticle formulation to study the cellular uptake of the nanoparticle in three cell lines, MCF-7, MDA-MB-231 and MCF-10A. MCF-7, MDA-MB-231 cell lines were seeded in 6-well plates (2×10^5^/well) and incubated with 250 μg/mL of coumarin 6-NP for 30, 60, 120 and 240 minutes. At the end of each incubation timepoint, cells were washed 3 times with PBS, harvested with trypsin and centrifuged for 5 minutes at 1100 rpm. Following supernatant aspiration, the cells were resuspended in 500 μL of PBS prior FACs analysis. Data were acquired on a Bio-Rad S3e Cell Sorter flow cytometer and analyzed using FlowJo software (Treestar). Representative FACS dot plots of gating strategy can be seen in the Figure S1. The population of cells was detected depending on their size and complexity (FSC-SSC gate) and later the doublets were excluded (FSC-Height/FSC-Area gate). Each experiment was performed in triplicate.

#### Fluorescence microscopy

To evaluate the cellular uptake of NPs in MCF-7, MDA-MB-231 and MCF-10A cell lines, coumarin 6, was used as a marker and was therefore encapsulated in the nanoparticles instead of res. Cells were seeded in 6-well plates at a density of 2×10^5^ cells per well with coverslips and incubated overnight to allow for cell attachment. Cells were treated with 250 µg/mL of coumarin 6-NP and incubated for 30 and 240 minutes. At the end of the incubation, cells were washed 3 times with PBS, fixed with 4% paraformaldehyde (PFA) for 10 minutes. Cells were washed 4 times with PBS. To quench auto-fluorescence coverslips were incubated with 1 M ammonium chloride for 15 minutes. Next, the coverslips were washed 3 times with PBS and mounted with DAPI mounting medium (Invitrogen/Thermo Fisher Scientific, Grand Island, NY USA). Fluorescence images were acquired on a Zeiss Axio Observer.A1 microscope. AxioVision and Photoshop CS6 software were used for image processing.

#### MTT assay

A total of 5×10^4^ cells/mL were seeded per well of a 96-well plate and incubated overnight to allow for cell attachment and recovery. At the end of each incubation period, cells were treated with different concentrations of res-loaded nanoparticles for the concentrations and time points described in the figure legends. Cell viability was measured using the MTT 3-(4,5-dimethylthiazol-2-yl)-2,5-monotetrazolium bromide assay. At the end of each incubation period, 20 μL of MTT dye (1 mg/mL; Sigma St. Louis, MO) was added in each well and the plate was incubated at 37 °C for 4 hours. Subsequently, the plates were read on a microplate reader (Wallac, PerkinElmer, Massachusetts, USA) at 570 nm. Absorbance was proportional to the number of viable cells per well. Percentage of cell viability in each group was calculated after normalization to its own control. All data are presented as mean values±standard deviation and representative for at least two independent experiments performed in triplicates.

### Statistical analysis

The data were expressed as the mean ± standard deviation. P values were calculated by the one-way ANOVA test and at the 0.05 level were considered statistically significant. Data analysis was carried out by GraphPad Prism 8.0 (GraphPad Software Inc., San Diego, CA, USA) and OriginLab OriginalPro v. 8.5.1.

## Results and Discussion

### Synthesis and characterization of NPs

Res was encapsulated by Pluronic F127 to improve its water solubility and enhance its stability, thereby improving its pharmacological potential. Additionally, coumarin 6 was encapsulated in the same manner to serve for diagnostic purposes. Both nanoparticles were prepared by a modified single emulsion method 46 using dichloromethane (DCM) as the solvent and vitamin E TPGS as the emulsifying agent. Vitamin E TPGS is a superior stabilizing agent offering improved emulsification, superior encapsulation efficiency as well as rendering proapoptotic properties thereby adding to the anticancer capability of the nanoparticle. The resulting nanoparticle forms via the spontaneous self-assembly of Pluronic F127 at or above its CMC of around 0.1% (w/v) 47-49. Pluronic F127 is an interesting block copolymer for use as a carrier for cancer applications because of its known anticancer potential 31-34,38. The resulting structure as illustrated in **Figure 1** consists of a core-shell arrangement. The hydrophobic inner core carries the drug and involves the PPO hydrophobic segment of the polymer and the tocopherol segment of Vitamin E TPGS. The hydrophilic shell which surrounds the core is made of the hydrophilic PEO segment of the polymer and the PEG chains of Vitamin E TPGS. The hydrophilic shell renders the nanoparticle water soluble and enables the delivery of the hydrophobic drug in biological media. This carrier system is unique because all its constituents are non-toxic, biocompatible and biodegradable. Additionally, most of the constituents have shown promising anti-cancer properties. To the best of our knowledge this is the first time such a configuration of materials has been combined into a single platform.

**Figure 3:**
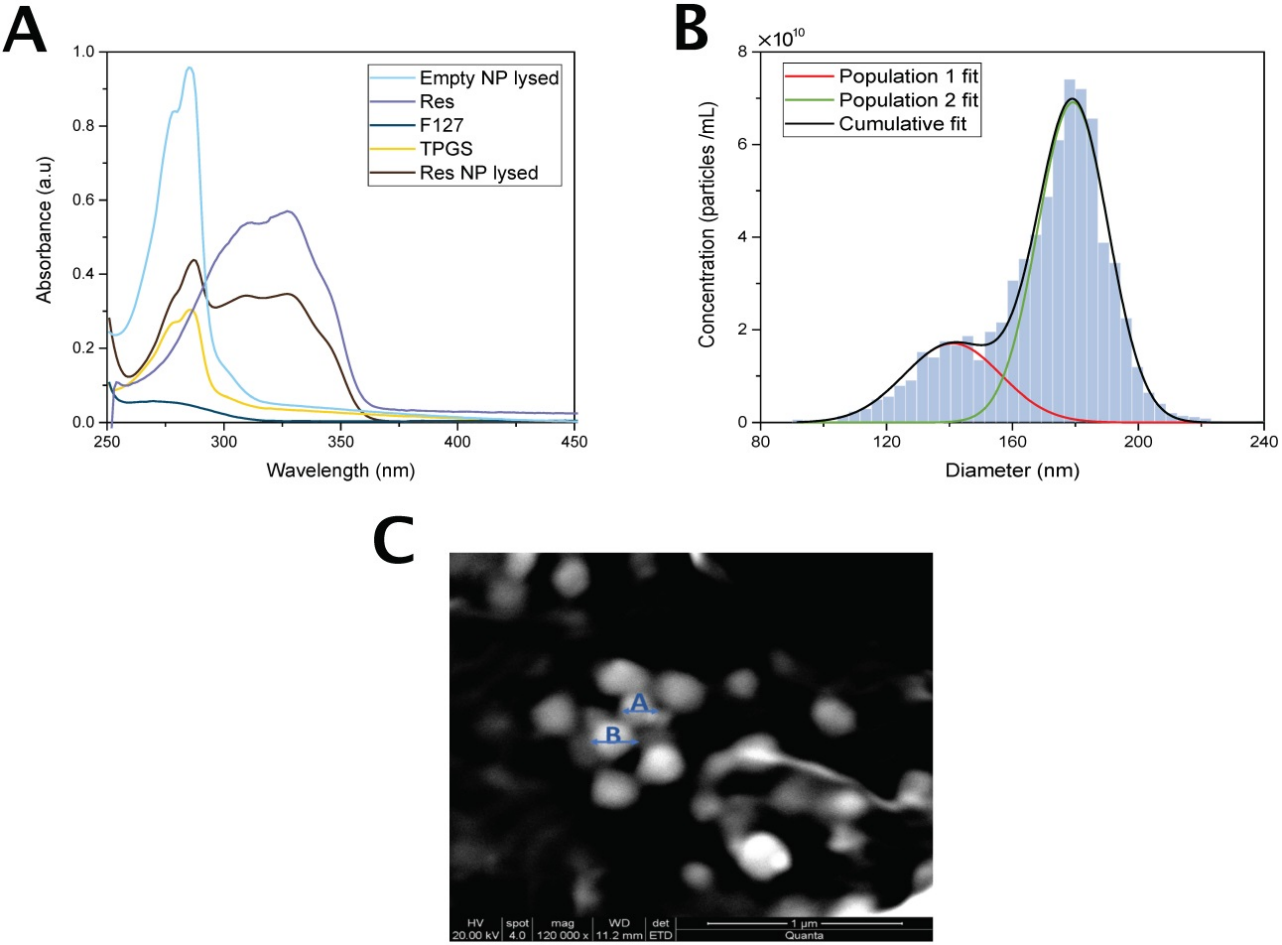
** Nanoparticle characterization schematic**. **(A)** UV-vis spectra of res, Pluronic F127, Vitamin E TPGS, Res-NP and Empty NP. Notes: Res shows a characteristic absorption peak at around 318 nm. Abbreviations: Res, resveratrol; Res-NP, Resveratrol loaded nanoparticle; and Empty-NP, empty nanoparticle. **(B)** Particle size distribution obtained from TRPS analysis of res-loaded nanoparticles. Solid lines: Gaussian distribution fits of two nanoparticle populations detected (red and green) and cumulative particle size distribution (black). Abbreviations: Res-NP, Resveratrol loaded nanoparticle. **(C)** Scanning electron microscopy (SEM) images of res-loaded NPs. The scale bar is 1 µm and the magnification during imaging was 120,000×. A and B are two indicative particles with sizes of 140 nm and 179 nm, respectively.

The content of the nanoparticle was verified by UV-Absorption Spectroscopy. **Figure 3A** shows the UV spectra of Pluronic F127, res and Vitamin E TPGS as well as the spectra of res-loaded and empty nanoparticles. Res-loaded nanoparticles exhibit an absorbance peak at 318 nm characteristic of res which is absent in the empty nanoparticle spectrum confirming the presence of res in the nanoparticle.

### Nanoparticle characterization

#### Size analysis by TRPS

The size and mean concentration of the nanoparticles were determined by TRPS and are shown in Figure 3B. Res-loaded nanoparticles appear to possess two size populations. The mean particle diameter of the most frequently occurring nanoparticles was 179±22 nm and that of the second frequently occurring was 140±30 nm. The measured mean concentration was 7.24×10^10^ particles/mL. The mean particle diameter of coumarin 6 loaded nanoparticles was 144±39 nm and the measured mean concentration was 2.73×10^10^ particles/mL. The similarity in size between the two types of nanoparticles suggests that coumarin 6 was a good model reporter for res.

The dependency of nanoparticle size with nanoparticle physicochemical characteristics is well established in the literature 50-52. Size determines the overall therapeutic efficacy of the nanoformulation by regulating nanoparticle biodistribution and tumor penetration as well as cellular internalization. Moreover, size governs nanoparticle clearance from the blood and excretion from body. Over the past few decades a consensus has been reached in regards to the desired size for cancer applications. If only in view of size variances nanoparticles ranging from 100 nm to 200 nm are preferred due to their extended blood circulation and the relatively slow rate of uptake by the reticuloendothelial system (RES) allowing for enhanced therapeutics 53. Consequently, most clinically approved nanoformulations for cancer applications have sizes in the range of 100 to 200 nm 54,55. The particle sizes in both res-loaded and coumarin 6-loaded nanoformulations are between 100 nm and 200 nm rendering these newly synthesized nanoparticles suitable for drug delivery.

#### Morphology and size analysis by SEM

The morphology of res loaded nanoparticles was examined by SEM. As shown in **Figure 3C** the nanoparticles are homogeneous and exhibit a spherical shape. The size as determined by SEM was estimated to be 184.42±38 nm.

#### Determination of the Encapsulation Efficiency and Drug loading content

The entrapment efficiency was found to be 73±0.9 % and the drug loading content was 6.2±0.1%. The entrapment efficiency and the drug loading content are important parameters to consider when accessing the nanomedicine's therapeutic effect and function. The entrapment efficiency is indicative of the extent of encapsulation of the drug in feed during the fabrication process and informs of the success of the entrapment 56. This parameter is mainly related to the preparation method of the nanomedicine and the amount of drug added during synthesis. Although the mechanism of entrapment was not investigated in this work, this high EE value could be attributed to the hydrophobicity of res and the interactions between res and the hydrophobic PPO segment of the polymer as well as with the aromatic ring of Vitamin E TPGS inside the core of the nanoparticle 57. The drug loading content on the other hand mirrors the ratio of drug to the entire nanoconstruct, demonstrating the percentage of mass of the nanoparticle that is owed to the encapsulated drug 45. The DL content is highly dependent on the physical and chemical properties of the carrier polymer. Generally speaking a drug loading content of less than 10% is considered low 58-60.

### Characterization of nanoparticle uptake in breast cancer cell lines using coumarin 6 as a model drug

#### Cellular Uptake by Flow Cytometry

**Figure 4:**
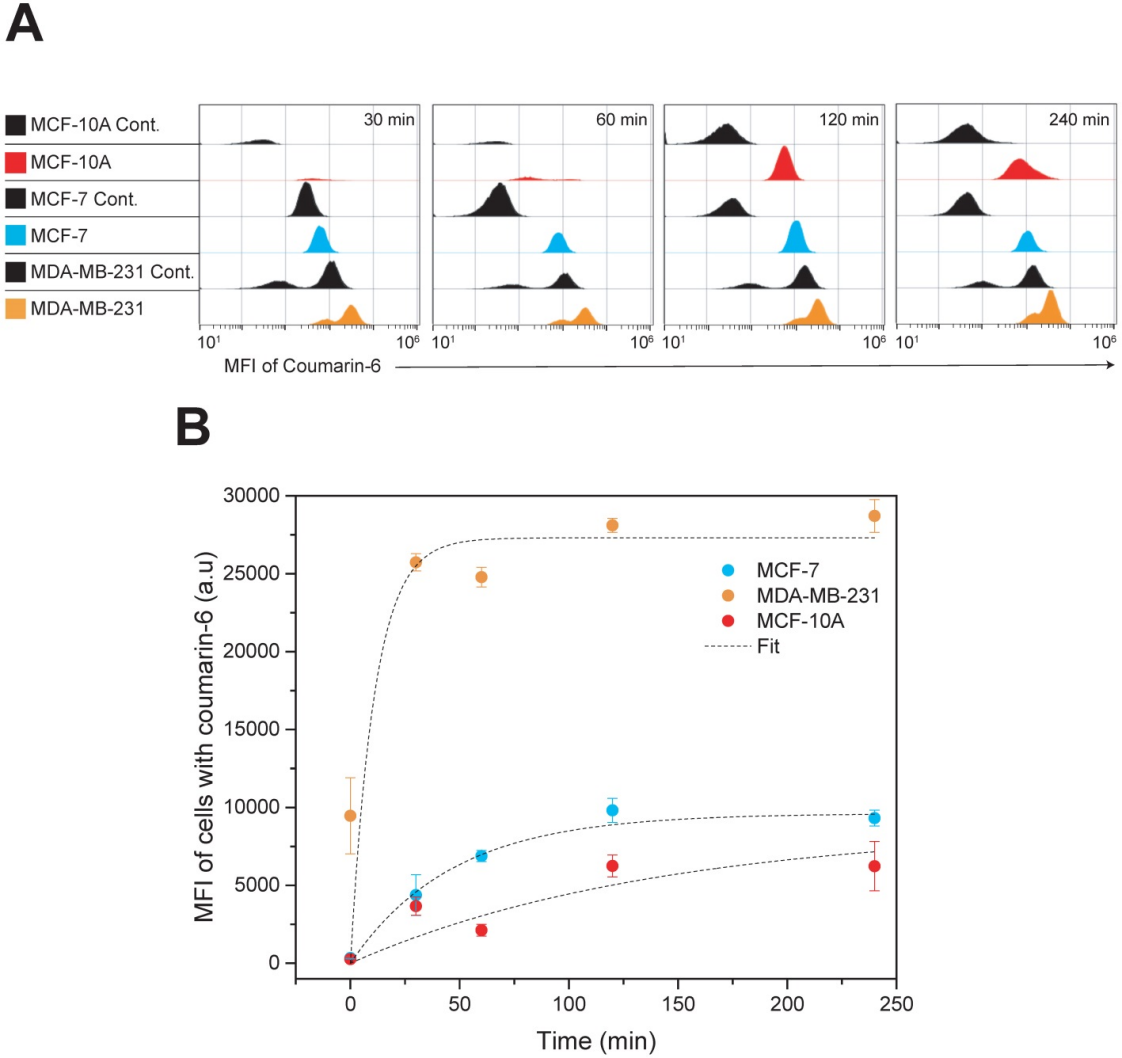
**Time dependent cellular uptake analysis by flow cytometry**. MDA-MB-231, MCF-7 and MCF-10A cells were incubated with Coumarin-6 loaded nanoparticles for up to 4 h.** (A)** Histograms of all cell lines at the given treatment groups showing the MFI of coumarin 6; MCF-7 cells (blue), MCF-10A (red) and MDA-MB-231 (orange). All controls are shown in black **(B)** Time kinetics of nanoparticle uptake in MDA-MB-231, MCF-7 and MCF-10A cell lines. Results are represented as mean MFI value ± SD, n=3. Data were analyzed by Origin Pro and fitted with Box-Lucas function Abbreviation: Mean Fluorescence Intensity (MFI).

Quantitative analysis of nanoparticle uptake kinetics of fluorescent nanoparticles was evaluated by flow cytometry and the results are presented in **Figure 4.** Coumarin 6 was used as the fluorescent model drug. The concentration of nanoparticle used was calculated taking the whole nanoparticle weight into account and corresponds to approximately 2.5 µg/mL of drug. Three cell lines, MDA-MB-231, MCF-7 and MCF-10A were incubated with the nanoparticles and their fluorescence was quantified by flow cytometry at various time points up to 4 h of incubation. To investigate the nanoformulations' differential uptake in transformed vs. non-transformed cells we used two invasive breast carcinoma cell lines (MDA-MB-231 and MCF-7) and non-malignant breast epithelial cells (MCF-10A). Both types are invasive breast carcinoma cells but possess various phenotypic/genotypic differences. Mainly, MCF-7 are hormone dependent while MDA-MB-231 are triple negative. The quantitative analysis by flow cytometry was very promising indicating that nanoparticle uptake in breast cancer cells was more effective compared to that in normal cells - with the TNBC cell line, MDA-MB-231, showing the most dramatic uptake. The Box-Lucas function, y=Y_o_(1-e^-bx^) was used to fit the data, where y is the Mean Fluorescence Intensity (MFI), x is time (min), Y_0_ is the maximum MFI (a.u) and b=1/τ. More specifically, MDA-MB-231 display a time constant (τ) of 11 min and a Y_0_ of 27,300 (a.u), MCF-7 display a time constant of 46 min and a Y_0_ of 9,618 (a.u) and MCF-10A display a time constant of 140 min and a Y_0_ of 8,748 (a.u). These results suggest specificity against cancer cells and the potential to reduce side effects if administered *in vivo*. Although the mechanism of entry was not examined in this study, a possible explanation for this difference in nanoparticle uptake between cell lines could be attributed to the ability of Pluronic block copolymers to induce changes in the microviscosity of cell membranes 38,61. Melik-Nubarov et al. 38 reported that these changes in the microviscosity are thought to occur as a result of the adsorption of the block copolymers on the cell membrane which consequently induced alterations in the membrane's structure. Most importantly, the alterations in membrane structure varied depending on whether the cell was cancerous or not. In fact, the effect was opposite with certain Pluronics increasing the microviscosity of cancerous cells but decreasing the microviscosity ('solidified') of the membrane of normal cells. Additionally, membrane fluidization in cancerous cells causes inhibition of the Pgp efflux function 62. This effect is exaggerated in our case due to the presence of Vitamin E TPGS which is known to do the same. Consequently, the enhanced uptake kinetics in MDA-MB-231 cells compared to MCF-7 could be explained due to higher Pgp expression in this type of cancer cell line 63. The enhanced nanoparticle internalization capacity seen in MDA-MB-231 cells is promising considering the limited treatment options for this specific type of cancer.

### *In vitro* cellular uptake

**Figure 5:**
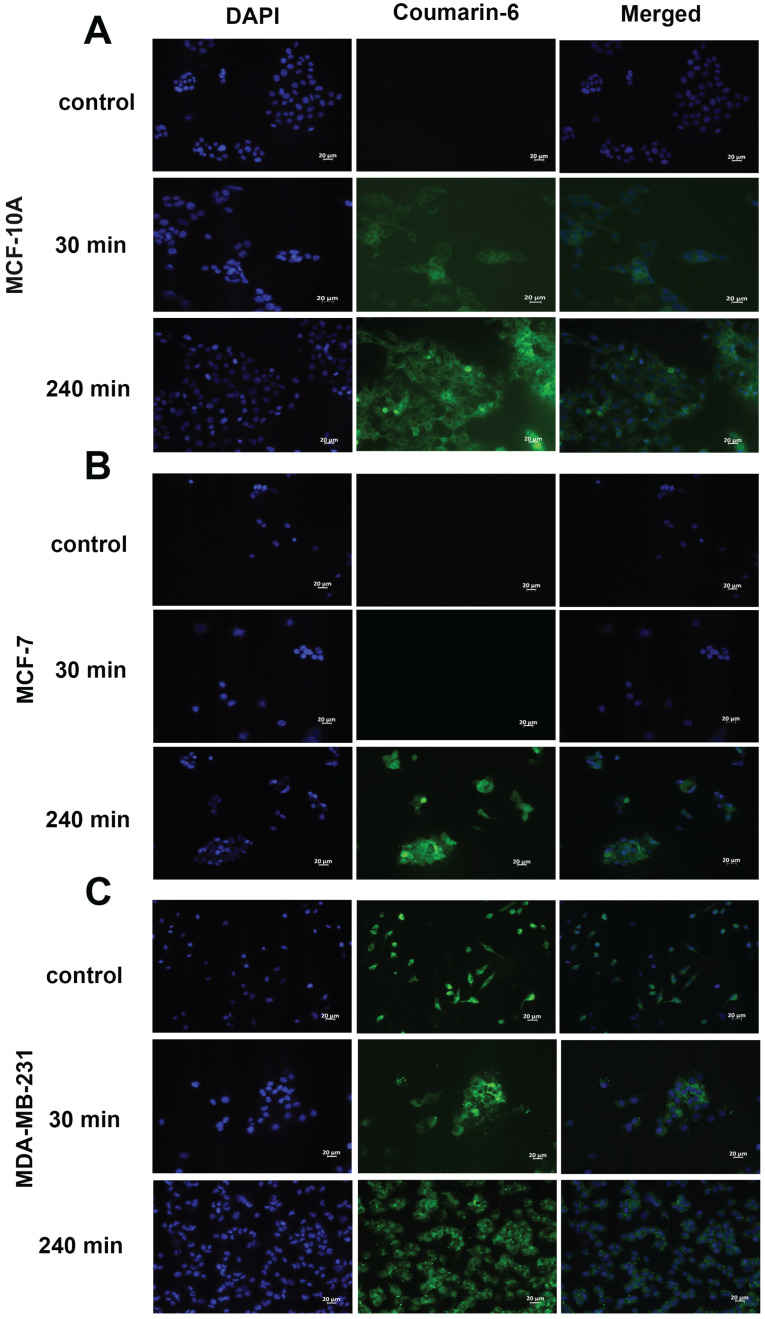
** Fluorescence microscopy of cells treated with coumarin-6 loaded nanoparticles.** In all panels blue represents nuclear staining (DAPI) and green represents coumarin-6 at 20× magnification. MCF-10A, MCF-7 and MDA-MB-231 cells were treated with 250 µg/mL of coumarin 6 NPs for 30 minutes and 240 minutes. Green fluorescence was observed in the cytoplasm around the nuclei of cells, suggesting uptake of coumarin 6 loaded NPs. Scale bar: 20 µm.

To qualitatively assess the uptake of nanoparticles by MCF-10A, MCF-7 and MDA-MB-231 cell lines, fluorescence microscopy was used. The cells were incubated with the nanoparticles loaded with coumarin 6 for 30 min and 240 min. The fluorescent images, as seen in **Figure 5**, clearly indicate nanoparticles are taken up into the cytoplasm of all cell lines at 4 h. Uptake was not seen at 30 min in MCF-7 (**Fig. 5B**) cells, contrary to the trend seen in flow cytometry. Additionally, morphological changes were observed in MDA-MB-231 cells upon treatment with res-loaded NPs. The MDA-MB-231 cell line changed from a spindle shaped morphology as seen in the control to a more spherical morphology at 30 and 240 min (**Fig. 5C**).

### Selective decrease in cell viability of breast cancer cell lines by res-loaded nanoparticles

**Figure 6:**
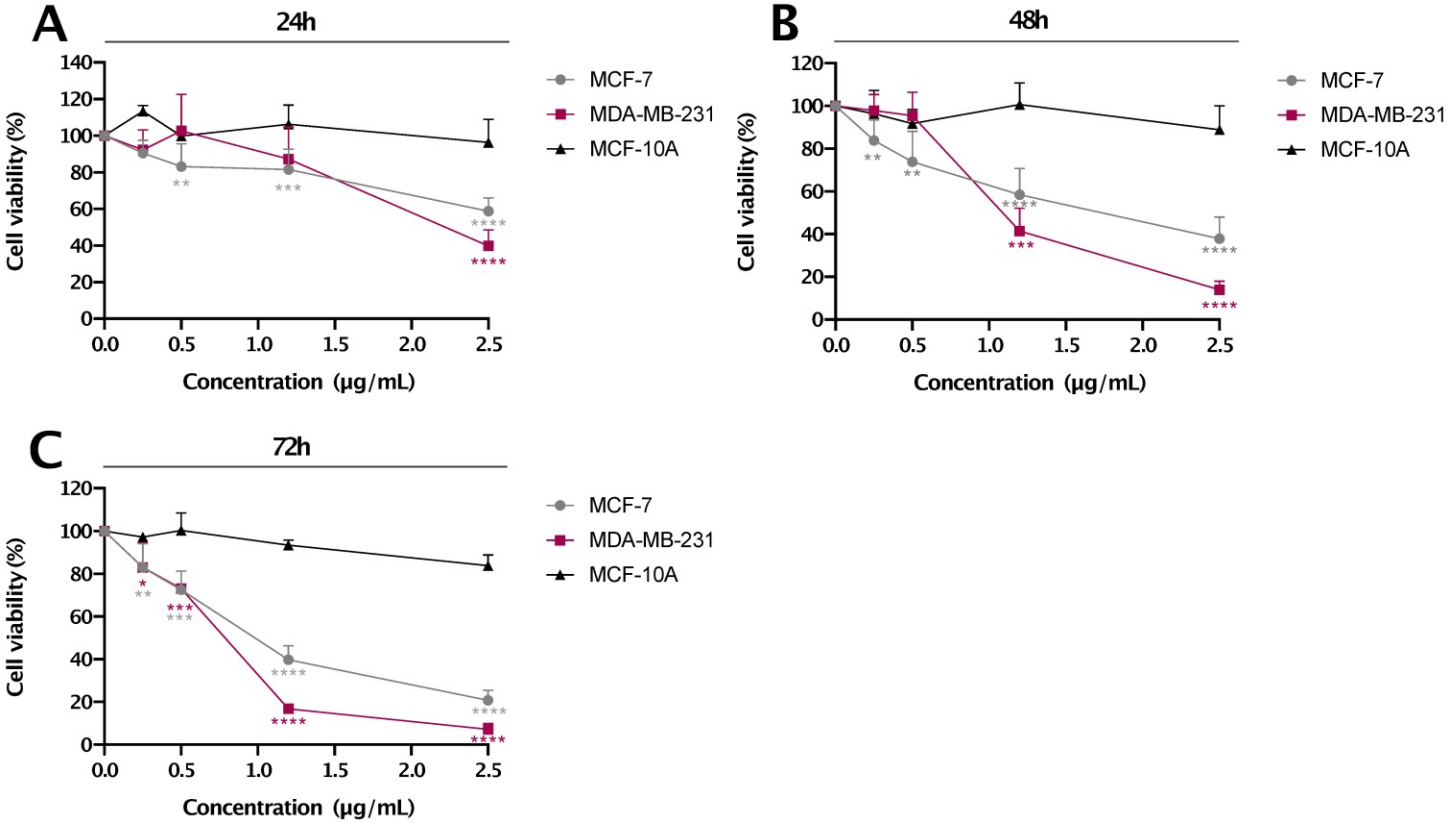
** The % cell viability vs. concentration (µg/mL) plot for res-loaded nanoparticles.** The anti-proliferative effect of res-loaded nanoparticles on MCF-10A, MCF-7 and MDA-MB-231 cell lines was examined at concentrations and timepoints as indicated: **(A)** treated for 24 h, **(B)** treated for 48 h and **(C)** treated for 72 h. All data are presented as mean values±standard deviation and are representative of at least two independent experiments performed in triplicate. *P* values: * <0.05, ** <0.01, *** <0.001, ****<0.0001 relative to control.

MCF-7, MDA-MB-231 breast cancer and MCF-10A immortalized breast cell lines were treated with different concentrations of res-loaded nanoparticles (as indicated in **Figure 6**) for 24, 48 and 72 h. The concentrations presented were calculated based only on the amount of res encapsulated in the nanoparticle. As shown in **Figure 6**, res-loaded nanoparticles were more effective and specific in reducing cell proliferation of MDA-MB-231 and MCF-7 cancer cell lines, and most importantly, with no significant effects on the 'normal' MCF-10A cell line. Specifically, at concentrations ranging from 0.5-2.5 μg/mL, res-loaded nanoparticles induced a marked reduction on cell viability of MDA-MB-231 and MCF-7 cell lines. Specifically, at a concentration of 2.5 μg/mL, MDA-MB-231 cell viability dropped to 39.8 % at 24 h, 14.0% at 48 h and 7.1% at 72 h, while the MCF-7 cell viability reduced to 58.7% at 24 h, 37.9% at 48 h and 20.7% at 72 h. The IC_50_ values are shown in **Table 1.** On the contrary, we did not observe any significant effects on the MCF-10A cell line at all concentrations and timepoints tested, which agrees with the previous results observed using flow cytometry. The *p* values can be seen in Table S1.

## Conclusion

In this study, we have successfully prepared and characterized a drug delivery system made from Pluronic F127 and Vitamin E TPGS for the encapsulation of res and coumarin 6, producing a fully soluble drug formulation. The resulting nanoparticle was effective at selectively targeting aggressive forms of breast cancer with no significant uptake by immortalized healthy epithelial cells. Additionally, the nanoparticle showed higher reduction in cell viability of breast cancer cells with no significant toxic effect on immortalized breast cells. These results suggest that the proposed nanoparticle is a promising platform for delivering drugs to breast cancer cells with a dual purpose: diagnosis and treatment.

## Supplementary Material

Supplementary figures and tables.

## Figures and Tables

**Figure 2:**
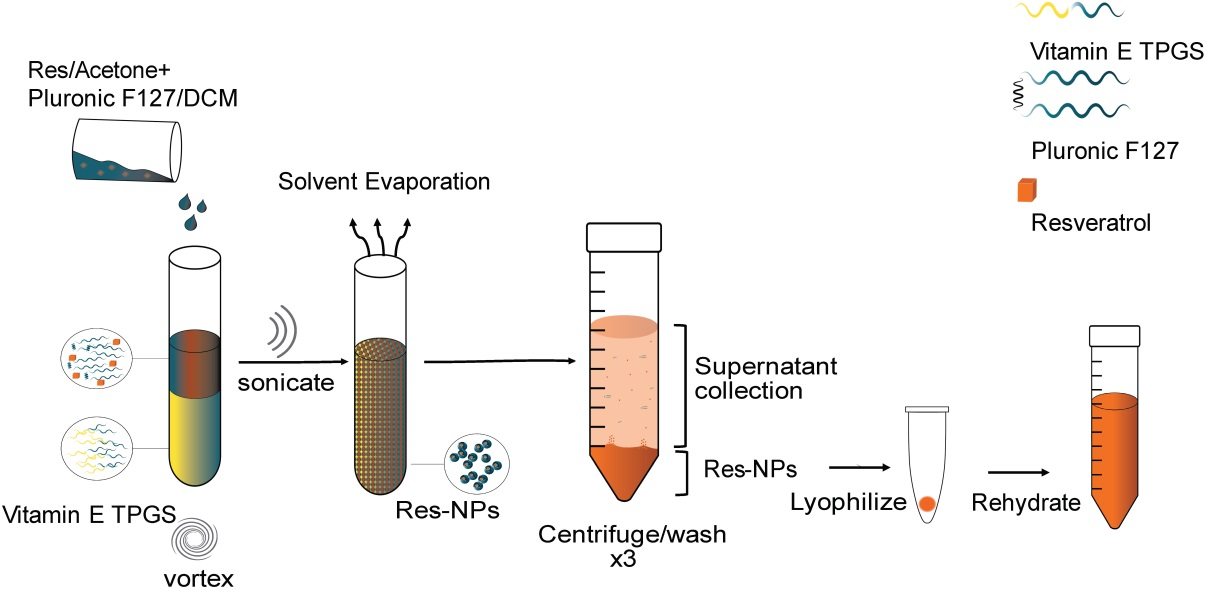
**Schematic illustration for the synthesis of resveratrol (res) loaded nanoparticles.** Res in acetone is mixed with dichloromethane (DCM) and then added to Vitamin E TPGS under vortex. The resulting mixture is ultrasonicated and the solvent evaporated under stirring. The resulting hardened nanoparticles are centrifuged and washed with d.H2O three times to remove unentrapped drug, Vitamin E TPGS and Pluronic F127. The supernatant is collected for use in further characterization studies. The pellet is collected and lyophilized to obtain NPs in solid form. The lyophilized NPs are rehydrated before use.

**Table 1:** ** IC50 values of res-loaded nanoparticles for all cell-lines at 24 h, 48 h and 72 h.** IC_50_ values were calculated using the GraphPad Prism 8.0 (GraphPad Software Inc., San Diego, CA, USA). N/A is used in cases where cell viability did not reach 50%

IC_50_			
	24 h	48 h	72 h
MDA-MB-231	2.32±0.12	1.06±0.12	0.76±0.04
MCF-7	N/A	1.61±0.57	0.93±0.09
MCF-10A	N/A	N/A	N/A

## Abbreviations

CMC: critical micelle concentration
DCM: dichloromethane
DL: Drug Loading
EE: Encapsulation Efficiency
ER: estrogen receptor
FBS: fetal bovine serum
MFI: Mean Fluorescence Intensity
MTT: 3-(4,5-dimethylthiazol-2-yl)-2,5-diphenyltetrazolium bromide
PEO: poly (ethylene oxide)
PFA: paraformaldehyde
PgR: progesterone receptor
PPO: poly (propylene oxide)
SEM: scanning electron microscopy
TNBC: triple negative breast cancer
TRPS: tunable resistive pulse sensing
Vitamin E TPGS: D-alpha-tocopheryl polyethylene glycol 1000 succinate
