# Supplementary material for: Resveratrol loaded polymeric micelles for theranostic targeting of breast cancer cells
Source: Nanotheranostics. 2021 Jan 1;5(1):113–24. doi: 10.7150/ntno.51955 (PMC7738945; doi:10.7150/ntno.51955)
Supplement: Supplementary file 1 — Supplementary figures and tables. [file ntnov05p0113s1.pdf]

Supplementary Information

Resveratrol loaded polymeric micelles for theranostic targeting of breast cancer cells

Authors: Yiota Gregoriou[1,2,3]*, Gregoria Gregoriou[1], Vural Yilmaz[1], Konstantinos Kapnisis[4], Marianna Prokopi[4], Andreas Anayiotos[4], Katerina Strati[1], Nikolas Dietis[5], Andreas I. Constantinou[1] and Chrysafis Andreou[2,3]*

[1] Department of Biological Sciences, Faculty of Pure and Applied Sciences, University of Cyprus, Nicosia, Cyprus

[2] *Department of Electrical and Computer Engineering* University of Cyprus, Nicosia, Cyprus

[3] Emphasis Research Centre, University of Cyprus, Nicosia, Cyprus

[4] Department of Mechanical Engineering and Material Science and Engineering, Cyprus University of Technology, Limassol, Cyprus

[5] Medical School University of Cyprus, Nicosia, Cyprus

* Corresponding Author: Yiota Gregoriou, Laboratory of Cancer Biology and Chemoprevention, Department of Biological Sciences, Faculty of Pure and Applied Sciences, University of Cyprus, P.O. Box 20537, 1678 Nicosia, Cyprus. E-mail: gregoriou.panayiota@ucy.ac.cy

* Corresponding Author: Chrysafis Andreou, Department of Electrical and Computer Engineering, University of Cyprus, P.O. Box 20537, 1678 Nicosia, Cyprus. E-mail: andreou.chrysafis@ucy.ac.cy

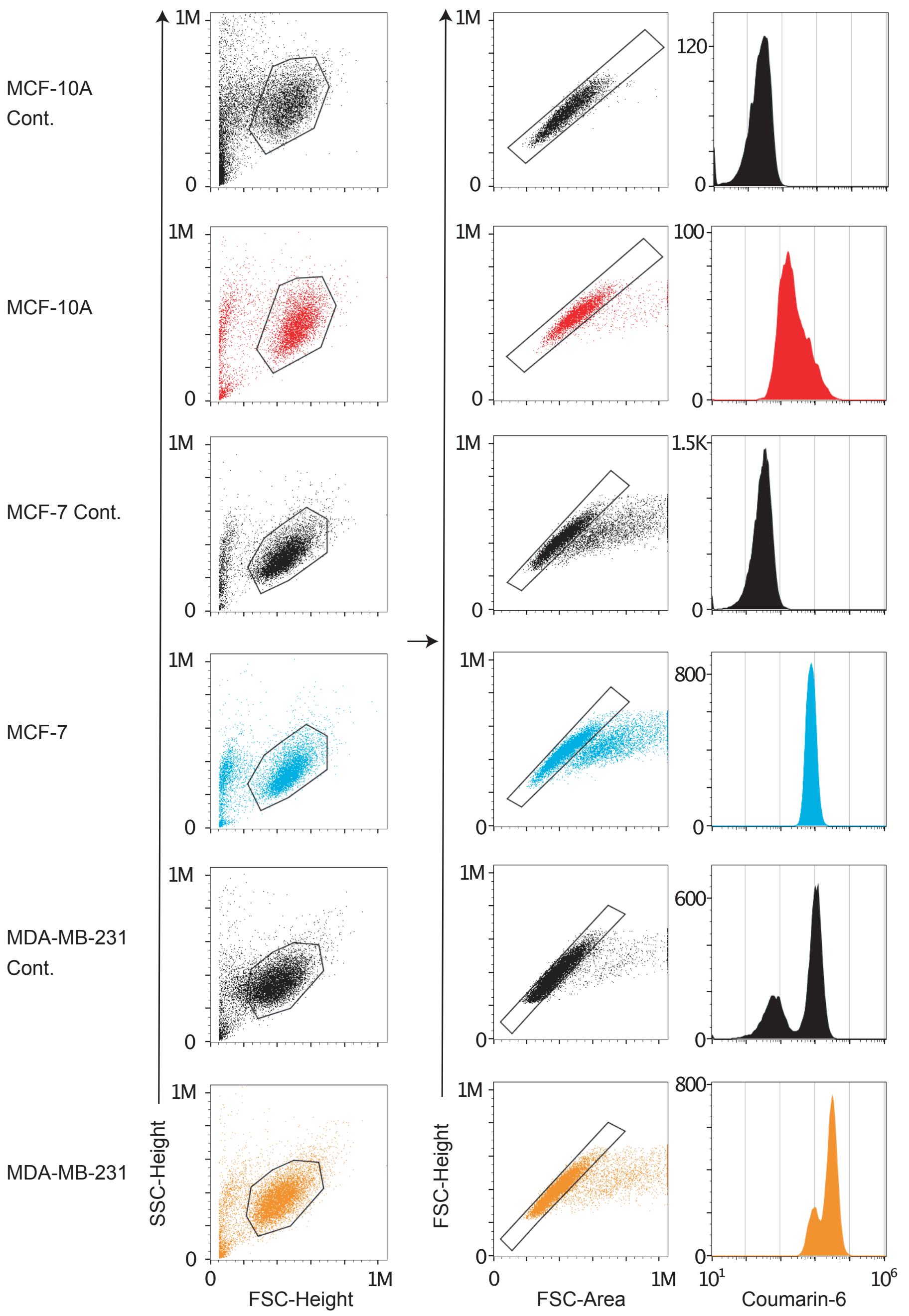

Figure S1. Representative FACS dot plots of gating strategy. Mean fluorescence intensity (MFI) of Coumarin 6 for all cell lines tested in the one-hour treatment group. The population of cells was detected depending on their size and complexity (FSC-SSC gate) and later the doublets were excluded (FSC-Height/FSC-Area gate). The Flow cytometric data from single cells is displayed as histograms.

P-value			
MDA-MB-231			
	24 h	48 h	72 h
0.25 µg/ml	ns	ns	0.0113
0.50 µg/ml	ns	ns	0.0003
1.2 µg/ml	ns	0.0001	<0.0001
2.5 µg/ml	<0.0001	<0.0001	<0.0001

P-value			
MCF-10A			
	24 h	48 h	72 h
0.25 µg/ml	ns	ns	ns
0.50 µg/ml	ns	ns	ns
1.2 µg/ml	ns	ns	0.0283
2.5 µg/ml	ns	ns	ns

P-value			
MCF-7			
	24 h	48 h	72 h
0.25 µg/ml	ns	0.0098	0.006
0.50 µg/ml	0.0016	0.0036	0.0002
1.2 µg/ml	0.0006	<0.0001	<0.0001
2.5 µg/ml	<0.0001	<0.0001	<0.0001

Table S1: P values of MCF-10A, MCF-7 and MDA-MB-231 cell lines. The values were calculated by the one-way ANOVA test and at the 0.05 level were considered statistically significant.
